# Aboriginal experiences of cancer and care coordination: Lessons from the Cancer Data and Aboriginal Disparities (CanDAD) narratives

**DOI:** 10.1111/hex.12687

**Published:** 2018-04-24

**Authors:** Rachel Reilly, Jasmine Micklem, Paul Yerrell, David Banham, Kim Morey, Janet Stajic, Marion Eckert, Monica Lawrence, Harold B. Stewart, Alex Brown, the other CanDAD Investigators and the CanDAD Aboriginal Community Reference Group

**Affiliations:** ^1^ Wardliparingga Aboriginal Research Unit South Australian Health and Medical Research Institute Adelaide SA Australia; ^2^ Infection and Immunity Aboriginal Health South Australian Health and Medical Research Institute Adelaide SA Australia; ^3^ Centre for Population Health Research University of South Australia Adelaide SA Australia; ^4^ School of Nursing and Midwifery Division of Health Sciences University of South Australia Adelaide SA Australia; ^5^ Poche Centre for Indigenous Health and Wellbeing Flinders University Adelaide SA Australia

**Keywords:** Aboriginal health, cancer, care coordination, disparities, qualitative research

## Abstract

**Background:**

Aboriginal people with cancer experience worse outcomes than other Australians for a range of complex and interrelated reasons. A younger age at diagnosis, higher likelihood of more advanced cancer or cancer type with poorer prognosis, geographic isolation and cultural and language diversity mean that patient pathways are potentially more complex for Aboriginal people with cancer. In addition, variation in the quality and acceptability of care may influence cancer outcomes.

**Objective:**

This study sought to understand how care coordination influences Aboriginal people's experiences of cancer treatment.

**Methods:**

Interviews with 29 Aboriginal patients or cancer survivors, 11 carers and 22 service providers were carried out. Interviews were semi‐structured and sought to elicit experiences of cancer and the health‐care system. The manifest content of the cancer narratives was entered onto a cancer pathway mapping tool and underlying themes were identified inductively.

**Results:**

The practice of cancer care coordination was found to address the needs of Aboriginal patients and their families/carers in 4 main areas: “navigating the health system”; “information and communication”; “things to manage at home”; and “cultural safety”.

**Conclusions:**

The CanDAD findings indicate that, when the need for cancer care coordination is met, it facilitated continuity of care in a range of ways that may potentially improve cancer outcomes. However, the need remains unmet for many. Findings support the importance of dedicated care coordination to enable Aboriginal people to receive adequate and appropriate patient‐centred care, so that the unacceptable disparity in cancer outcomes between Aboriginal and non‐Aboriginal people can be addressed.

## 1 INTRODUCTION

The Cancer Data and Aboriginal Disparities (CanDAD) project was developed in response to the significant and increasing disparity in cancer mortality between Aboriginal and other Australians in South Australia (SA).^1^, ^2^ Studies conducted nationally indicate that the drivers of this disparity are likely to be varied and include a higher rate of exposure to risk factors including but not limited to smoking, lower uptake of cancer screening and higher rates of comorbidity.^1^, ^3^ Once diagnosed, Aboriginal people face challenges within the health system stemming from language diversity and differences, racism, poor interagency coordination, cultural misunderstandings, emotional and physical stress, travel, financial problems and isolation, which can make the experience of receiving treatment for cancer emotionally distressing, inefficient and potentially dangerous.^4^, ^5^, ^6^, ^7^

For several reasons, the treatment needs of Aboriginal people with cancer are often greater and more medically complex than those of their non‐Aboriginal counterparts. Aboriginal people are more likely to be diagnosed with cancers with poorer prognosis.^8^ Cancers of the liver, gallbladder, head and neck, oesophagus and lung are relatively more common among Aboriginal people, while melanoma, breast and prostate tumours are relatively less common.^9^, ^10^ These cancers are also commonly detected at later stages, more metastasized or disseminated cancer at diagnosis compared to non‐Aboriginal people of the same age, sex and cancer type.^10^, ^11^ Added to this are complications from comorbid diabetes and other chronic diseases, which are more common in Aboriginal than non‐Aboriginal patients.^1^

**Figure 1:**
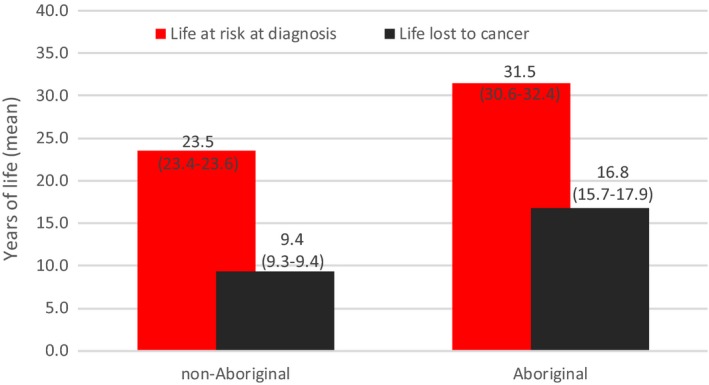
Life at risk and life lost from cancer, South Australia, 1990‐2010 . Source: CanDAD NHMRC Partnership Project

Focusing on SA specifically, epidemiological analysis carried out for the CanDAD project has identified a range of disparities in outcomes that warrant consideration when tailoring health services to meet the needs of this population. For example, in line with national data, Aboriginal people are generally younger when diagnosed with cancer, with a difference in median age of diagnosis of 10 years (58 vs 68 years).^9^ The scale of the disparity may be more fully appreciated when expressed as years of life affected by cancer.^12^, ^13^ For a non‐Aboriginal person in SA, cancer diagnosis at 65 threatens a further 24 years of life expectancy (Figure 1), while for an Aboriginal person in SA, typically diagnosed at a younger age, the life expectancy at risk is one‐third higher at 32 years.^14^ Overall, an Aboriginal person in SA is far more likely to die prematurely because of cancer.^1^, ^10^

Over half the Aboriginal people diagnosed with cancer in SA live in outer regional and remote regions.^9^ Consequently, the degree to which they can benefit from cancer treatments is limited by a lack of local services (including for screening and early detection).^9^, ^14^ Long distance travel is often necessary to access specialist treatment, leading to experiences of isolation from community, as well as language and cultural differences.^4^ These difficulties are reflected in the epidemiological data with those living in remote locations at the time of diagnosis being significantly more likely to die within 5 years of that diagnosis compared to those living in a major city (31.1% vs 48.2%, *P* < .001).^9^ These disparities are difficult to understand and address without a deeply contextualized qualitative understanding of cancer experiences.

In recognition of the potential complexity of patient pathways for Aboriginal people with cancer and the unacceptable cancer disparity, a statewide Aboriginal cancer forum held in 2006^15^ recommended the introduction of dedicated Aboriginal Cancer Care Coordinators to the major metropolitan public hospitals in SA. In response, 2 part‐time senior nursing positions were funded in 2011 for a period of 12 months, with iterative cycles of short‐term programme funding enabling the continuation of the positions to the time of writing. Care coordination is intended to facilitate continuity of care by providing assessment, management and on‐going review of clinical and supportive care needs to Aboriginal patients from the time of notification to attend hospital, at admission, throughout treatment and follow‐up.^16^ This includes continuity of care from country to hospital and return home. These positions continue to operate under insecure funding arrangements. Efforts to adequately evaluate the roles are confronted with the challenge of describing the complex and varied tasks that together make up care coordination.

While there is no consensus definition in the international literature for “care coordination” across disease groups and health settings, it has been defined broadly as, “the deliberate organization of patient care between 2 or more participants involved in the patient's care to facilitate the appropriate delivery of health‐care services”.^17^ International evidence provides some support for the utility of such roles for minority populations, including Indigenous populations. In the United States, studies have shown that “patient navigators” provide important emotional support and practical assistance for both mainstream^18^ and Indigenous populations.^19^ With “insider” knowledge of both the patient's social and cultural circumstances and of the health‐care system, patient navigators also help to overcome the mistrust that prevents or delays engagement with the medical system.^20^

When implemented in Australia for eye and cardiac care, dedicated care coordination for Aboriginal people has effectively decreased non‐attendance, reduced wait times, increased clinical throughput and improved communication, discharge planning and satisfaction among patients and their families.^21^, ^22^ In addition, the implementation of dedicated care coordination has reduced incidences of delayed or cancelled surgery.^22^ A recent consensus statement from the National Heart Foundation for overcoming disparities between Aboriginal and non‐Aboriginal people in the management of acute coronary syndromes presents an argument for Aboriginal cardiac care coordinators to be incorporated into the health‐care system to liaise with service providers; review and monitor patients; communicate with families; ensure cultural factors are considered when organizing care; and to address social issues as patients move through the health‐care system.^21^, ^23^

With a view to improving the response of the health‐care system to the needs of Aboriginal people, the CanDAD project aims to address disparities in cancer care by developing a comprehensive cancer monitoring and surveillance system capable of integrating epidemiological and narrative data. The qualitative component of CanDAD sought to understand the experiences of Aboriginal people as they interact with the health system. This included identifying gaps in care and exploring the impact of the various components of current cancer care provision (including care coordination) on Aboriginal patients’ pathways from multiple perspectives. Here, we present findings from the CanDAD narratives relevant to the question of how care coordination, provided by dedicated care coordinators or others, addressed gaps in care or otherwise influenced Aboriginal people's experiences of cancer care.

## 2 METHODS

### 2.1 Participants

Participants were Aboriginal people with experience of cancer as patients, carers or family members and Aboriginal and non‐Aboriginal service providers. Participants were referred to the project by Aboriginal Cancer Care Coordinators at a major metropolitan hospital in SA; Aboriginal Community Controlled Health Services in multiple regions of SA; and snowball sampling.

### 2.2 Data collection

Face‐to‐face, semi‐structured interviews were carried out between January 2015 and July 2016 by a team of interviewers comprising male, female, Aboriginal and non‐Aboriginal researchers. Having provided informed consent, participants nominated their preferred interviewer and location, with a view to maximizing cultural safety. Interpreters were made available, but none were used as participants were proficient in English as a first or additional language. The interview guide was developed following review of relevant literature and input from senior Aboriginal cultural advisors and researchers (HS, KM, JS and AB). Interviews took between 40 and 90 minutes, were audio‐recorded, professionally transcribed and deidentified prior to analysis.

### 2.3 Data analysis

Data were analysed in 2 ways. The manifest content of the narratives was entered onto a “cancer pathway mapping tool.”^24^ The adaptation and use of the tool have been described elsewhere.^2^ The tool provided a practical way to record multiple stakeholder perspectives of where gaps and failures occurred for Aboriginal people, relative to an “optimal” cancer care pathway as defined by current health system policies.^25^ Underlying influences on the overall patient pathway were identified as latent themes in thematic analysis carried out using N‐Vivo 11.3.0. Themes were identified and refined in iterative cycles of revision with Aboriginal cultural advisors in partnership with Aboriginal and non‐Aboriginal researchers (RR, JM and PY). The project was approved by the SA Health and Aboriginal Health Council of SA Human Research Ethics Committees and was overseen by an Aboriginal community reference group comprising senior Aboriginal people with experience of cancer representing a cross section of geographic regions and cultural groups in SA.

## 3 RESULTS

Participants ranged in age from 19 to 75. Twenty‐nine were Aboriginal patients or cancer survivors (55% Male), 11 carers (64% female) and 22 service providers, (77% female). Eight service providers were non‐Aboriginal. Non‐identifying participant characteristics are shown in Table 1. Cancers of concern to patients and carers were of the following: respiratory system (n = 6); gastrointestinal system (n = 4); breast (n = 4); head and neck (n = 3); reproductive organs (n = 4); leukaemia and lymphoma (n = 7); and others (n = 3).

**Table 1:** Participant demographics

	Sex		Residence			Age group				Recruitment		
	Male	Female	Urban	Regional	Remote	18‐25	26‐45	46‐65	66+	ACCC	Health Service	Snowball
Patients	16	13	11	3	15	5	2	13	6	19	1	9
Carers	4	7	2	5	4	1	5	5	0	3	2	6
Service Providers	5	17	13	3	6					4	14	4
Total	25	37	26	11	25	5	2	13	6	26	17	19
%	40	60	42	18	40	10	11	29	10	42	27	31

### 3.1 Findings

Within the patient pathways and underlying themes, “care coordination” was identified when service providers proactively sought to facilitate the continuity of care. This was achieved by proactively identifying and finding solutions to a range of gaps in care or other barriers faced by patients and their carers/families. Care coordination was carried out by the metropolitan‐based Aboriginal Cancer Care Coordinators, generalized care coordinators or patient pathway officers in suburban, regional or remote health services and hospitals, and lastly, in the absence of dedicated care coordinators, by nursing and other staff at smaller health services and hospitals who took on care coordination as part of their role.

**Figure 2:**
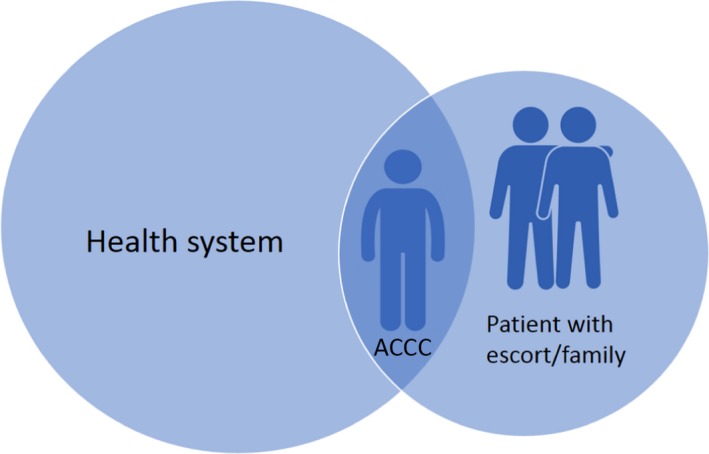
The Aboriginal Cancer Care Coordinator providing care informed by “insider” knowledge of the health system and of the social and cultural needs of the patient

The findings supported the notion that care coordination operates at the interface between the health system and Aboriginal community and requires some “insider” knowledge of both, as well as the ability to engage appropriately and effectively in both settings (Figure 2).^20^ As such, care coordination requires an in‐depth knowledge of cancers, their treatments and the health system as well as Aboriginal community protocols and social systems.

**Figure 3:**
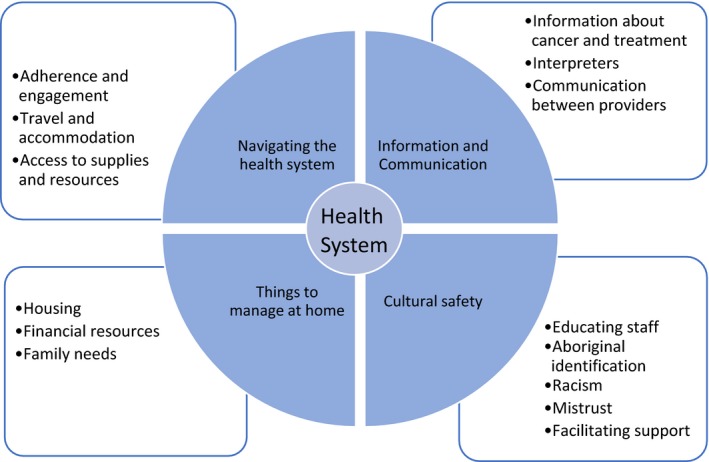
The key areas where care coordination influenced patient pathways identified as themes in the CanDAD narratives

From this unique position, care coordination addressed the needs of Aboriginal patients in 4 main areas: “navigating the health system,” “information and communication,” “things to manage at home” and “cultural safety”. Ways in which care coordinators acted to address gaps in care within each of these areas are summarized in Figure 3 and described in more detail below. To maintain the confidentiality of the participants, direct quotes are labelled: “SP” (service provider); “AP” (Aboriginal people with current or past experience of cancer); or “AC” (carers, escorts or family members of Aboriginal people with cancer).

#### 3.1.1 Navigating the health‐care system

##### Promoting adherence and sustaining engagement

Care coordination was particularly relevant for patients with more complex cancer diagnoses or psychosocial circumstances, for example, patients who needed to travel for treatment, whose families were unavailable to support them or whose diagnosis required input from multiple service providers. Where this complexity was recognized and understood, care coordinators could harness the potential flexibility within the system:

*…there's flexibility within the system and I think it needs somebody to…encourage staff to think flexibly. If somebody can't come for their treatment for a period, to advocate for things to be put on hold and then restarted. [Otherwise] they might've just thought, no, we're going to just finish the treatment because this person hasn't come*. *[SP1]*

Care coordinators also worked to help patients understand the need for treatment and to keep track of appointments to avoid becoming disengaged from treatment. In some cases, this required assertive outreach:

*…the [metropolitan health service] rang me and said, “Can you get onto her, can you talk to her, we're really worried she's missing this and that.” So, I go out…and I say, “Hey, look, you've got to go back. You've got to keep up with your treatment otherwise you get sick again.” And she says, “Oh, true.”…So she started going back again*. *[SP17]*

##### Travel and accommodation

Organizing travel, accommodation and supportive care needs for patients and their families was a key component of care coordination. Travel was often an expensive burden for patients and their families, added to the cost of having to take time off work for treatment. When care coordination support was absent, some families described resorting to inappropriate or uncomfortable options, such as taking long bus trips while in severe pain or having nowhere to stay:

*Spent my whole pay, $1200 ‘cause it was an expensive flight out of the blue …My sister flew in the same morning and her other half …We all met at the hospital, we were there for 1 week without accommodation. We were in the waiting room sleeping with all our swags ‘cause we had no‐one to organise accommodation at that time*. *[AC4]*

With their knowledge of the Patient Assistance Transport Scheme (PATS) and other resources available to families, care coordinators could arrange travel for treatment or for family members to accompany patients. Care coordinators were known for this among other service providers:

*The father was ringing us for 3 days ‐ “I'm scared,” you know, it wasn't so much for the client it was that the father was scared. And, so, that's when I made contact with [care coordinator]. I said, “You need to get them here.”* *[SP3]*

The services available for patient transport were described as complicated and often mismatched to the reality of people needing to travel urgently on low incomes:

*What patient needs to…hear PATS saying, “Well, that's not my problem, they need to plan and save their money.”? A lot of the Aboriginal people have different deductions coming out of their pension money and by the time they get it in their hands some of them would be lucky to have $170 a fortnight. Now, you try and buy an air ticket…for $170 a fortnight*. *[SP17]*

In response, care coordinators would spend time ensuring that paperwork was completed to enable patients to travel. This required liaising with patients and doctors:

*…so I rang back to the specialist and I said…”These people have had that [transport] claim rejected now because of you not doing your job.” I said, “Can you fill it in properly and send the form back?” Took me 3 weeks…* *[SP17]*

##### Access to supplies and resources

Facilitating access to supplies and resources for patients and their families entering the hospital was an early care coordination task. Through experience, coordinators learnt that they needed to ask questions, as patients “*don't always volunteer information.” [SP4]*.

*Have you got warm clothing with you? Do we need to source you a jacket or something? The escorts ‐ have they got their medication with them?…* *[SP4]*

Upon discharge, care coordinators supported patients to access the resources they needed to go home, such as appropriate medications. At times, this required insider knowledge of the health system and funding schemes. In one health‐care setting, patients would be charged for medications if they were provided by the hospital pharmacy, but not if they had their prescription filled by their local pharmacy:

*… I mean, people are prescribed new medications while they're here or and it can be quite expensive. Instead…the pharmacist will fax it to the local pharmacy. So when assistance can be given it is*. *[SP1]*

Care coordinators provided an opportunity for patients and their carers to deal with a single person, instead of having to manage multiple providers. As explained here:

*Our head's not even screwed on, we're not thinking straight. We'd rather deal with 1 person, that's what [care coordinator] does. We deal with her and she tells anyone to sort anything out for us and then it gets sorted. Where in [Interstate hospital] it was‐we had 10 million people coming in and introducing themselves and giving us cards. I've got a purse full of cards from [Interstate hospital], don't know who they are*. *[AC4]*

#### 3.1.2 Information and communication

##### Information about cancer and treatment

For several patients, care coordinators played an important role in hearing and understanding information about prognosis and assisting with treatment choices. Absorbing information about cancer and its treatment can be difficult, especially when in shock or when there may be more pressing issues to think about:

*… Because everything else takes precedence, if you've got no money, no food, your housing's up the creek, you've got people coming and going and family and, you know, things happening with kids. And then getting into the cancer business…if you don't know what they're talking about and you go in by yourself, it just goes straight over*. *[AP25]*

Importantly, care coordinators offered support during early consultations, to ensure that the patient and their partners or families understood the information they were provided, enabling them to make informed treatment choices.

*[The care coordinator] is good. Especially in them first 7 weeks. She was here every appointment I come to. She come in when…they first said lymphoma. Cos sometimes you know, they'd talk and it would just go over your head… And, she come in there when they told me…12 months [pause] life expectancy. So [she] was there, she was really good*. *[AP2]*

Having another person there who can interpret the information also provided reassurance for some patients:

*[The care coordinator]'s been…She's been a rock.… She comes in with me just in case I miss something and explains everything to me … and then she'll say, ‘Well, I wouldn't worry about that,’ you know, she reassures you*. *[AP13]*

##### Interpreters

There were multiple examples of miscommunication or poor communication, leading to: a sense of alienation, patients not wanting to return to the health service or acting on information they later discovered was inadequate or misinterpreted.

*Pitjantjatjara, Yankunytjatjara, Pitjantjatjara ‐ that's their first language… they don't understand what the doctor's saying because you haven't got a lot of people that speak our language in the hospital here… A lot of them go‐go back and they end up passing on because they don't really understand it*. *[AP1]*

Access to interpreters was frequently described as extremely limited for Aboriginal patients:

*Oh, there's an interpreting coordinator at the hospital…And they do every single language except Aboriginal*. *[SP24]*

Unfortunately, interpreting was too often left to family members or other service providers, who may not be adequately skilled to fulfil this role:

*I don't think that they're utilising interpreters as well as they should. Well they're probably using the health worker. But I'm not sure how that's working and whether they're utilising them as they should be. And you know, are they trained? Are the nursing staff trained to work with Aboriginal interpreters to get the information across clearly? And to communicate with the client in a proper way?* *[AP29]*

To address this clear inadequacy, care coordinators would assist with finding interpreters where possible:

*And even today I go with people to specialist appointments, and these poor buggers they don't understand a word they're saying. That's why we go with them, to interpret what that person, that doctor, or that nurse is telling them*. *[SP8]*

##### Communication between providers

Care coordinators also facilitated communication between treatment teams both within and between hospitals to ensure treatment plans were coordinated:

*…we were able to go to a multi‐disciplinary team meeting and say, look somebody needs to make some decisions here, because you're all saying, ‘Refer to breast oncology, oh and hang on, see what ‘gynae onc’ want to do, hang on what are haematology wanting to do, and nobody was making a decision about anything.’ And in the end, a team finally agreed, yes, somebody has to take a lead here…* *[SP4]*

Coordinating care across rural and remote services was sometimes difficult, with many patients returning to places where access to supports and health services may be limited:

*I'm going back, but I'm only going back to my family. I'm not going back to any follow‐up support people back there, because they don't exist …As much as I'm excited to go home…it's also scary…I'm going home to a farm. There's no supports*. *[AP8]*

Ideally, care coordinators ensured that information reached patients’ primary health‐care services and assisted with planning return visits for follow‐up:

*So [we]’re not just like “See ya, we've finished with you now”…[We] spend a lot of time co‐ordinating that discharge ‐ particularly if you've got a mobile type …I'll say, “So you don't mind if I speak to your clinic?”* *[SP4]*

This process was easier when care coordinators in different locations shared information through established channels:

*…because you've got these cancer care coordinators in every step of the journey, you've got great flow of information happening, you've got the heads‐up that they're coming back … and treatment plans are relayed so that they're not lost in the system*. *[SP4]*

#### 3.1.3 Culturally safe care

##### Educating staff and aboriginal identification

In response to a lack of culturally sensitive practice, some care coordinators communicated cultural needs to doctors and other staff, demonstrating their unique position at the interface between the health system and Aboriginal community members (Figure 2). In this sense, the care coordinator role extended to providing cultural education to non‐Aboriginal staff. Identifying Aboriginal patients is the first step towards providing culturally appropriate care:

*Identification of Aboriginal people has always been a problem in this hospital. When [we] first started, we did…education with admin about asking the question, because a lot of people are a bit loathe to ever ask the question, “Are you Indigenous?” for some reason. So we did a lot of education…and there are posters now all over the place…* *[SP4]*

##### Racism and mistrust

Unfortunately, some participants recounted stories of overt racism within the health‐care system. For example:

*I went down to have x‐rays and there was a whole heap of orderlies sitting there around their waiting area. And I heard a racist remark by one of the orderlies. And I ended up in tears…It's like, oh Jesus, you're here crook and you hear a racist joke*. *[AP2]*

This incident was addressed with assistance from the cancer care coordinator, who supported the patient to respond:

*[The care coordinator] said, “Well, it's not acceptable”…And she didn't force me or anything, she said‐ “You need to really consider doing an official complaint.” So I thought, yeah, no, bugger it, I've got […] cancer and I'm hearing shit like that! So, yeah, I did an official report*. *[AP2]*

Racism was viewed as a barrier to Aboriginal people accessing health services at all:

*…a lot of people won't speak up because they don't want to go through all that rigmarole of the name calling and all that sort of stuff…so they shut up, they stay quiet and they don't talk unless it's to one of their own…they know is going to listen*. *[AC14]*

Mistrust of the health system was commonly expressed by participants and recognized as a barrier to accessing health services.

*… a lot of them get scared when they go into hospitals because when family members go into hospital all they do is die because they wait at home for so long before they go and see a doctor…* *[AC13]*

From the Aboriginal Cancer Care Coordinators’ perspectives, building trust is a priority:

*…the first and foremost thing…is having that trust; that I'm there, that I am listening to them…And I'm there to speak on their behalf, and at any stage on the journey…and I will advocate on their behalf to make sure they get the treatment that they should be getting*. *[SP4]*

##### Facilitating appropriate treatment and support

A lack of cultural safety within hospitals was attributed in part to a lack of staff who could speak Aboriginal languages or identify cultural issues and have them addressed appropriately. This could include gender issues:

*…he was embarrassed about white women washing him as a traditional man and he was having tears rolling down his face dealing with that…You try and tell white people that's not their culture, women shouldn't be seeing those things or touching…* *[SP17]*

Care coordinators also provided education to families so that they were not afraid to have their family member with cancer return home and assisting people to return to country at the end of life. As expressed by a past patient:

*… someone like [name of care coordinator]…saying, “You right there? You got somebody at home? Do you want me to come and visit you, or, do you want me to try and help family come and visit you?” because the family's too scared or they may not have ways and means to come and visit you*. *[AP19]*

For some patients, shame was preventing them from telling their families about their cancer diagnosis, and in response, care coordinators facilitated contact between Aboriginal people with cancer to try and reduce any stigma:

*…[the care coordinator]'s been asking people, some from rural backgrounds if they want to be available to talk to other Aboriginal people that are diagnosed with cancer, and they're happy to do it*. *[SP1]*

#### 3.1.4 Things to manage at home

##### Managing housing and finances

Aboriginal participants described multiple stressors competing with the management of cancer treatment. Understanding the impact of these competing pressures was viewed as an important component of care.

*Aboriginal people have so many more psychosocial issues and other issues besides the actual cancer diagnosis happening…If that's all known and people can be assisted with those things along with their cancer treatment, that really is important. Because that's when people stop coming to their treatment*. *[SP5]*

Participants described the need to deal with insurance, housing, social welfare or other agencies as a source of stress that was difficult to manage while also managing cancer treatment:

*Ultimately it would be great to have that 1 person come into your home and go off and sort it all out …someone…better equipped to give an objective view…to organise for your payslips to go to housing, and they sort out your new rent…* *[AP2]*

There were several examples where care coordinators provided this type of assistance—for example, by organizing for bills to be paid. However, it was acknowledged that this type of support was generally out of scope:

*Certainly, we make referrals to social work…They have more knowledge about those resources and that's not our role anyway, to assist with financial things. Although we do write letters, of course, for support for housing, and sometimes it's a matter of we do it because nobody else is doing it*. *[SP1]*

##### Family needs

Addressing the needs of families was integral to the role of care coordinators, because of the centrality of family to the wellbeing of patients. The separation of families when a caregiver had to leave home to receive treatment in the city was a burden for many:

*It's like‐because we've been with the kids ever since. And now with this here happening, and we are all like coming up and down. And it's just like they ‐…I don't know ‐ they mostly feel like lost or messed up inside, because we're here and they are back there. Because we've been all time together…* *[AP1]*

So in some cases, this meant caring for family members who are travelling with the patient:

*…it's problematic if people haven't got money to pay for it themselves. And if they need medications or they're crook themselves, which happens, we can arrange appointments for them. Yeah, but‐ the systems have always got criteria …And so when people don't meet those criteria, which happens regularly, then that's where we have to get a little bit creative and find other services. And that's really time‐consuming*. *[SP1]*

In many cases, care coordinators attempted to keep families together or address the needs of children left at home. The resources and services available to do so varied from case to case and, in many cases, were limited as follows:

*…if they're from the country and they want [the children] to come here and stay with them we often can't assist them, although we do what we can. …Sometimes they can come down short‐term…but funding has to come from the community or they need to find funding themselves…we can't actually provide the funding for that to happen*. *[SP1]*

In other cases, care coordinators supported family members to cope as they navigated the health system on another's behalf:

*My mum…received a lot of support from them [the care coordinators], but not so much me. I wasn't feeling very community minded or like I wanted any help or anything like that throughout it*. *[AP4]*

## 4 DISCUSSION

In line with previous reporting by Natale‐Pereira and colleagues,^20^ the CanDAD findings indicate that, when accessed, care coordination plays a critical role in addressing the social, cultural and logistical barriers to effective participation in the health‐care system that disproportionately burdens Aboriginal people, as well as providing education and support to enable patients to adhere to prescribed therapies. However, at present, care coordination occurs in the context of significant systemic and structural limitations. For example, a lack of facilities for families to accompany patients during treatment is a clear gap. Care coordinators are limited in how much funding they can access for patients, and there are currently very few accommodation or childcare options for families with children, particularly when longer periods of stay are necessary. Care coordinators attempt to facilitate transport options for patients within a system that is not agile and does not cater well for those on low incomes and to communicate important information to people with diverse language backgrounds with limited access to interpreters and other cultural brokers.

Nonetheless, the narratives demonstrate that, from diagnosis, through treatment including surgery, chemotherapy and radiation therapy and follow‐up, care coordinators consistently work to reduce the barriers to engaging with the health system, and thereby reduce the risk of negative outcomes that could otherwise stem from the following: mistrust of the health system, misunderstanding and miscommunication of information, loss to follow‐up and receiving culturally inappropriate care. The CanDAD findings suggest that care coordination for Aboriginal people with cancer is equally as valuable as similar roles established for other conditions. In the light of this, it is heartening that the current South Australian Aboriginal Cancer Control Plan (2016‐2021)^16^ highlights the continuation and expansion of care coordination as a priority area for action, alongside further action to improve the provision of supportive care to Aboriginal people with cancer and their families. However, care coordination is not available in all metropolitan hospitals, let alone in many regional locations, so the need remains unmet for many.

Care coordination is considered one of the core dimensions of patient‐centred care, alongside treating patients with dignity and respect; communication of appropriate information about their clinical condition and treatment options; and encouraging patient participation in decision making.^26^ Patient‐centred care is necessarily culturally competent, and these 2 approaches overlap in their essential features.^27^ Both in Australia and internationally, patient (or person)‐centred care is, in turn, considered one of the pillars of quality health care,^28^ and there is an increasing focus in the literature and within health systems on the necessity of understanding patients’ experiences of the health system to know whether the care provided is meeting appropriate standards of clinical safety and effectiveness.^29^, ^30^ This study gives voice to experiences of Aboriginal people in SA and supports the importance and value of a patient‐centred approach to health‐care delivery for disadvantaged groups.

### 4.1 Limitations

Participant recruitment by Aboriginal Cancer Care Coordinators could be seen to bias the data towards those who viewed them more favourably. This recruitment strategy was used because these care coordinators were ideally in contact with all identified Aboriginal people being admitted to the major metropolitan hospital for cancer treatment. When recruiting, the care coordinators were asked to assess whether a potential participant was well enough to take part in an interview and to refer them to the research team. In practice, this meant some participants were well known to the care coordinators, but several were not. It is worth noting that participants were also recruited via other health services and snowball sampling (Table 1).

The study employed a deductive approach to data analysis in the first instance, as a pragmatic attempt to provide feedback on the cancer care pathway as defined within contemporary health policies. Inductive coding identified underlying influences on this pathway. While this approach had the practical advantage of reflecting on the current health system, we acknowledge that it may have limited analysis, for example by preventing alternative definitions of “optimal care” from being fully explored. Lastly, this study, while providing strong evidence for the importance of care coordination for Aboriginal cancer patients and their families, does not constitute a formal evaluation. Such an evaluation, incorporating both qualitative and quantitative methods, is an important future step.

## 5 CONCLUSION

Findings indicate that dedicated care coordination has the potential to reduce disparities in cancer care by facilitating more effective interactions between Aboriginal people with cancer and the health‐care system. A clear message from the CanDAD narratives is that there is a need to further build and strengthen the Aboriginal workforce within the health system. While this workforce remains underdeveloped, the need for dedicated care coordination is particularly acute. Dedicated care coordination is an important step towards enabling Aboriginal people with cancer and their families to access their fundamental right to appropriate, patient‐centred care and to help address the unacceptable disparity in cancer outcomes between Aboriginal and non‐Aboriginal people.
